# *Acinetobacter nosocomialis*: Defining the Role of Efflux Pumps in Resistance to Antimicrobial Therapy, Surface Motility, and Biofilm Formation

**DOI:** 10.3389/fmicb.2018.01902

**Published:** 2018-08-21

**Authors:** Daniel B. Knight, Susan D. Rudin, Robert A. Bonomo, Philip N. Rather

**Affiliations:** ^1^Research Service, Atlanta Veterans Affairs Medical Center, Decatur, GA, United States; ^2^Research Service, Louis Stokes Cleveland Department of Veterans Affairs Medical Center, Cleveland, OH, United States; ^3^Department of Medicine, Pharmacology and Molecular Biology and Microbiology, Case Western Reserve University School of Medicine, Cleveland, OH, United States; ^4^Case Western Reserve University Veterans Affairs Center for Antimicrobial Resistance (Case-VA CARES), Cleveland, OH, United States; ^5^Department of Microbiology and Immunology, Emory University School of Medicine, Atlanta, GA, United States; ^6^Emory Antibiotic Resistance Center, Emory University School of Medicine, Atlanta, GA, United States

**Keywords:** *Acinetobacter*, RND-efflux, motility, biofilm, antimicrobial resistance

## Abstract

*Acinetobacter nosocomialis* is a member of the *Acinetobacter calcoaceticus-Acinetobacter baumannii* (ACB) complex. Increasingly, reports are emerging of the pathogenic profile and multidrug resistance (MDR) phenotype of this species. To define novel therapies to overcome resistance, we queried the role of the major efflux pumps in *A. nosocomialis* strain M2 on antimicrobial susceptibility profiles. *A. nosocomialis* strains with the following mutations were engineered by allelic replacement; Δ*adeB*, Δ*adeJ*, and Δ*adeB/adeJ*. In these isogenic strains, we show that the Δ*adeJ* mutation increased susceptibility to beta-lactams, beta-lactam/beta-lactamase inhibitors, chloramphenicol, monobactam, tigecycline, and trimethoprim. The Δ*adeB* mutation had a minor effect on resistance to certain beta-lactams, rifampicin and tigecycline. In addition, the Δ*adeJ* mutation resulted in a significant decrease in surface motility and a minor decrease in biofilm formation. Our results indicate that the efflux pump, AdeIJK, has additional roles outside of antibiotic resistance in *A. nosocomialis*.

## Introduction

*Acinetobacter nosocomialis* is a Gram-negative opportunistic pathogen that is grouped into the *Acinetobacter calcoaceticus-Acinetobacter baumannii* (ACB) complex (Nemec et al., 2011; Visca et al., 2011). The ability of *A. nosocomialis* to cause disease in humans is well-recognized (Wisplinghoff et al., 2012; Chusri et al., 2014; Huang et al., 2014), although studies suggest the virulence of this bacterium may be lower than the closely related bacterium *Acinetobacter baumannii* (Peleg et al., 2012; Lee et al., 2013; Yang et al., 2013; Fitzpatrick et al., 2015). Many potential virulence factors have been identified in *A. nosocomialis* and include a CTFR inhibitory factor (Cif), a protein O-glycosylation system, a type-I secretion system, a type-II secretion system, secretion of outer membrane vesicles, the OmpA protein, the CpaA protease, and quorum sensing (Niu et al., 2008; Bahl et al., 2014; Harding et al., 2015, 2016, 2017; Nho et al., 2015; Weber et al., 2015; Kim et al., 2016; Kinsella et al., 2017).

*A. nosocomialis* strain M2 was isolated in 1996 from a hip infection and has been extensively studied, particularly with respect to the virulence factors described above. M2 was formerly classified as *A. baumannii*, but whole genome sequencing resulted in its reclassification (Carruthers et al., 2013). While *A. nosocomialis* can be highly resistant to antibiotics, the role of RND-type efflux pumps in this process has not been investigated in this bacterium. Two primary efflux systems in the closely related *A. baumannii* are the AdeABC and AdeIJK efflux systems (Magnet et al., 2001; Damier-Piolle et al., 2008). Each efflux system is composed of an outer membrane channel (AdeC, AdeK), a membrane fusion protein (AdeA, AdeI) and an inner membrane transporter (AdeB, AdeJ). In addition to the efflux of antimicrobials, these systems can impact additional phenotypes in the cell, such as surface motility, biofilm formation, and virulence (Yoon et al., 2015; Richmond et al., 2016).

In this study, we investigated the role of AdeABC and AdeIJK orthologs in *A. nosocomialis*. Similar to what is observed in *A. baumannii*, loss of AdeIJK had a major impact on antibiotic susceptibility profiles. In contrast, the loss of AdeABC had a minimal impact on susceptibility. Interestingly, the loss of AdeIJK reduced surface motility, indicating additional roles for this RND-type efflux system in *A. nosocomialis*.

## Materials and methods

### Bacterial growth conditions, strains, and plasmids

*A. nosocomialis* strain M2 was used for all studies and has been described previously (Carruthers et al., 2013). *E. coli* strains EC100D and CC118 were used for general cloning. *E. coli* strain SM10 was used for bacterial conjugations. Growth media consisted of 10 g tryptone, 5 g yeast extract, and 5 g NaCl per liter. Agar was added at 15 g per liter. For sucrose counter-selections, media was prepared as described above, but without NaCl and containing 10% sucrose. Cloning vectors were pBC.SK- (Agilent) and pKNG101 (Kaniga et al., 1991).

### Construction of *adeB* and *adeJ* mutations

Internal fragments of the *adeB* and *adeJ* genes were obtained by PCR amplification of M2 genomic DNA using the following primers. peg93.for 5′- TTGCTAAGTATTCCTAAATTAC-3′ and peg93.rev 5′- TTAGGAAGAGATTTTTTTC−3′ for *adeB*, and peg1681.for 5′- ATGGCACAATTTTTTATTCATC−3′ and peg1681.rev 5′- TCACGATTTATGCTCCTGAG-3′ for *adeJ*. The resulting PCR generated fragments were cloned into the pBC.SK digested plasmid with SmaI, creating padeB and padeJ. The padeB plasmid was then digested with NarI, which digests once in the middle of the *adeB* gene and treated with T4 DNA polymerase to create blunt ends. This was then re-ligated to create a frameshift mutation in *adeB*. The plasmid padeJ was digested with SphI, which cuts once in the middle of *adeJ*, treated with T4 DNA polymerase to create blunt ends and re-ligated to create an *adeJ* frameshift mutation. The mutated *adeB* and *adeJ* genes were then excised as an XbaI-SalI fragment and cloned into the suicide vector pKNG101 digested with XbaI and SalI. Each plasmid was transformed into *E. coli* SM10 and then introduced into the *A. nosocomialis* M2 chromosome by conjugation. Exconjugants were grown for 10 generations in LB broth without antibiotic and dilutions were plated on lysogeny broth (LB) plates without sodium chloride and containing 10% sucrose. Colonies containing the *adeB* or *adeJ* frameshift mutations were identified by PCR amplifying each gene and the digesting the resulting PCR products with either NarI for *adeB* or SphI for *adeJ*. The presence of each chromosomal mutation was indicated by the failure of each enzyme to digest the fragment and each mutation was verified by DNA sequence analysis. To create an *adeB, adeJ* double mutant, the *adeB* mutant was used as the parent and the *adeJ* mutation was crossed into the chromosome as described above. To create an *adeB::Km* mutation, an EZ-Tn5 <Kan-2> insertion centrally located in the *adeB* gene present in pKNG101 was recombined into the chromosomal copy of *adeB* as described above.

### Antimicrobial susceptibility testing

*A. nosocomialis* strain M2 and its isogenic derivatives were subject to antimicrobial susceptibility testing using E-Test Strips, Trek, and MicroScan platforms. Additionally, disk diffusion assays were performed using Mueller Hinton agar for several antibiotics alone and in combination with boronic acid transition state inhibitor (BATSI) compounds SM23 and S02030 (Powers et al., 2014; Nguyen et al., 2016). For TREK, strains were tested once. For the disc diffusion and Etest assays, strains were tested in duplicate.

### Motility assays

The base media for motility assays consisted of 10 g tryptone, 5 g yeast extract, and 5 g NaCl per liter. Media was solidified using 0.35% Eiken agar (Eiken Chemical Ltd. Tokyo, Japan). Plates were used the same day they were prepared. For testing the motility of the M2 strain and various mutants, cultures were grown up to early log phase, adjusted to the same optical density of A_600_ = 0.15 by the addition of sterile LB broth and a 1 μl drop was placed on the center of the plate. Plates were incubated at 30°C and motility was measured after 14 h. Statistical analysis was done using the Student's *T*-test.

### Biofilm analysis

Cells for biofilm analysis were taken directly from freezer stocks and grown in 2 ml 0.5X LB without shaking at room temperature to an optical density A_600_ of 0.1. Each tube was then used to inoculate wells of a 96 well microtiter plate with 150 μl of culture. Plates were incubated stationary at 30 or 37°C for 24 h. The optical density of each well was read at A_600_ for cell growth and the planktonic cells were removed. To stain biofilms, 250 μl of 10% crystal violet was added to each well for 30 min. The crystal violet was gently decanted and each well was gently washed three times with distilled water. Three hundred microliters of 33% acetic acid was added to each well to solubilize the crystal violet and the absorbance of a 1/10 dilution was read at A_585_. Statistical analysis was done using the Student's *T*-test.

## Results

### Analysis of AdeABC and AdeIJK RND-Efflux systems in *A. nosocomialis*

*A. nosocomialis* strain M2 contains orthologs of AdeA, AdeB and AdeC that share 94, 98, and 92 percent amino acid identify, respectively, to the corresponding proteins in *A. baumannii* strain AB5075.UW. In addition, orthologs of the AdeIJK proteins were found with 97, 99, and 98 percent identity to the corresponding proteins in *A. baumannii* AB5075.UW. To investigate the function of each RND-type efflux system, null alleles in the *adeB* and *adeJ* genes, encoding the inner membrane transporter for each system were constructed by introducing frameshift mutations in each gene into the chromosome by allelic replacement (section Materials and Methods).

The antibiotic susceptibility profile of each mutant was then determined for a panel of antibiotics representing different classes (Table 1). The loss of *adeB* had a minimal effect on the overall levels of resistance and a slight increase in susceptibility was observed for ampicillin, cefotaxime, amikacin, rifampin, and tigecyline (Table 1). This result was surprising as the AdeABC system has a prominent role in antibiotic resistance in *A. baumannii* (Magnet et al., 2001). To determine if this *adeB* frameshift mutation was somehow being suppressed or was not a null allele, we constructed an *adeB::Km* mutation, where the *adeB* gene was disrupted in the middle of the coding region. However, this *adeB::km* mutant displayed the same level of resistance to ampicillin (128 μg/ml), tetracycline (2 μg/ml), and ciprofloxacin (0.38 μg/ml) as wild-type, indicating that the previously isolated frameshift mutation in *adeB* was non-functional.

**Table 1:** Antimicrobial susceptibility profiles.

	**M2 wild-type**	**M2 *ΔadeB***	**M2 *ΔadeJ***	**M2 *ΔadeB, ΔadeJ***
**E-TEST (mg/L)**				
Ampicillin	24	16	8	8
Cefotaxime	>32	24	1.5	1.5
Ceftriaxone	>32	>32	3	3
Chloramphenicol	64	64	6	6
Amikacin	4	3	3	3
Rifampin	12	8	3	3
Tigecycline	0.25	0.19	0.032	0.023
Trimethoprim	>32	>32	1.5	1.5
**TREK (mg/L)**				
Piperacillin/tazobactam	16/4	≤ 8/4	≤ 8/4	< 8/4
Ceftazidime	4	4	≤ 1	≤ 1
Cefuroxime	16	16	≤ 4	≤ 4
Amikacin	≤ 4	≤ 4	≤ 4	≤ 4
Aztreonam	>16	>16	4	4
Meropenem	< 1	< 1	< 1	< 1

The effect of a mutation in *adeJ* on antibiotic susceptibility was far more pronounced, where cells became more susceptible to the following antibiotics; ampicillin (3-fold), cefotaxime (>15-fold), ceftriaxone (>10-fold), chloramphenicol (>10-fold), rifampin (4-fold), tigecycline (8-fold), and trimethoprim (>20-fold) (Table 1). The antibiotic susceptibility profiles were also examined for an *adeB/adeJ* double mutant to determine if the loss of both efflux systems had additional effects. However, the *adeB/adeJ* double mutant essentially phenocopied the *adeJ* single mutant (Table 1).

We next assayed the role of AdeB and AdeJ efflux pumps in the handling of the boronic acid transition state inhibitors (BATSIs) SM23 and S02030 (Powers et al., 2014; Nguyen et al., 2016). These BATSIs either mimic the acylation or deacylation transition state. Paired with a penicillin (ampicillin), carbapenem (meropenem), or cephalosporin (ceftazidimne or cefepime) as performed herein, the BATSI can act to inhibit serine based beta-lactamases *in-vitro*. As a result of this mechanism of action, class C cephalosporinases possess the greatest affinity for these compounds (e.g., ADC cephalosporinase in *A. nosocomialis*). Our results indicate that the BATSI studied are substrates for the AdeIJK efflux pump in *A. nosocomialis* (Table 2). In particular, the susceptibility of wild-type M2 to cefotaxime is unaffected by these inhibitors, but in the presence of the *adeJ* mutation, these inhibitors now increase susceptibility to cefotaxime (Table 2).

**Table 2:** Disk diffusion results (zone size in mm).

**Antibiotic (mg/L) + inhibitor (mg/L)**	**M2**	**M2, *ΔadeB***	**M2, *ΔadeJ***	**M2 *ΔadeBΔadeJ***
Ampicillin 10	12	12	18	18
Ampicillin 10 + SM23 10	15	15	21	21
Ampicillin 10 + S02030 10	15	14	19	19
Ceftazidime 10	18	18	23	23
Ceftazidime 10 + SM23 10	18	18	23	24
Ceftazidime 10 + SM02030 10	18	19	24	23
Cefotaxime 10	16	16	24	24
Cefotaxime 10 + SM23 10	16	17	27	26
Cefotaxime 10 + S02030 10	16	16	26	25
Meropenem 10	24	24	30	30
Meropenem 10 + SM23 10	25	25	30	30
Meropenem 10 + S02030 10	24	24	30	29

### Role of AdeABC and AdeIJK in motility

**Figure 1:**
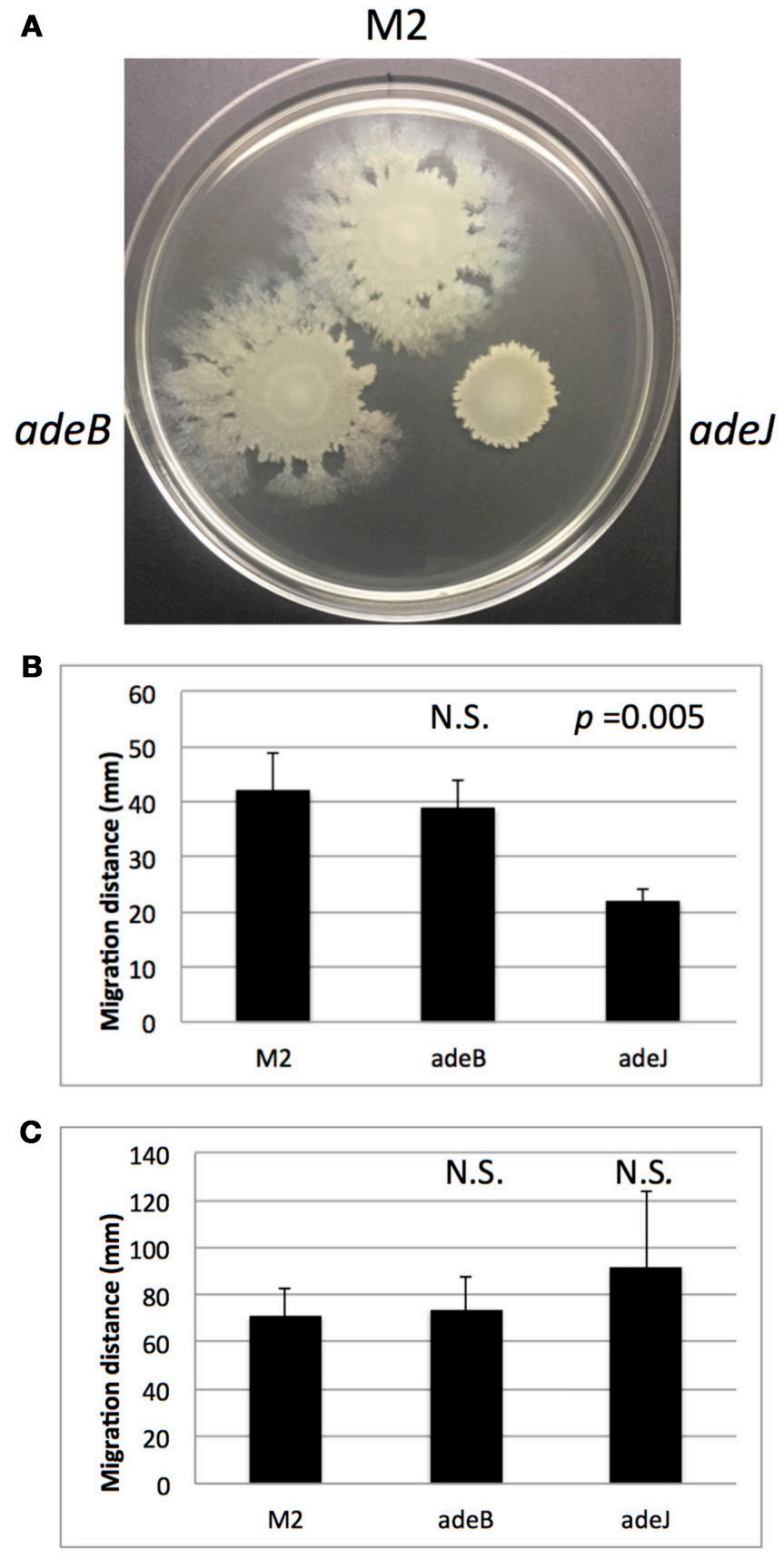
Surface motility of wild-type M2 and efflux mutants. Wild-type M2 and the isogenic *adeB* and *adeJ* mutants were assayed for surface motility as described in the Materials and Methods. In **(A)**, motility of the indicated strains is shown after 14 h at 30°C. Motility was quantitated from 4 separate experiments at 30°C **(B)** and 37°C **(C)**. Error bars represent standard deviation of the mean. N.S. indicates a *p*-value > 0.05.

*A. nosocomialis* strain M2 is capable of rapidly translocating across soft agar surfaces (Clemmer et al., 2011). Although the mechanism responsible for this motility is unclear, a number of genes have been identified that reduce motility including mutations in the *abaI* autoinducer synthase responsible for quorum sensing signal production (Clemmer et al., 2011). We tested the wild-type M2 parent and the isogenic *adeB* and *adeJ* mutants for their motility phenotypes at 30 degrees. The *adeB* mutation did not significantly alter surface motility (Figures 1A,B). In contrast, the *adeJ* mutation had a pronounced effect on surface motility, with a greater than 50% reduction relative to the wild-type M2 parent (Figure 1). Interestingly, this motility defect was temperature dependent, at 37 degrees the *adeJ* mutant exhibited a similar level of motility as wild-type (Figure 1C).

In a previous study, the motility of *A. nosocomialis* M2 was shown to be dependent on production of the quorum sensing signal 3-OH C_12_-HSL (Clemmer et al., 2011). To investigate the possibility that the motility defect in the *adeJ* mutant was due to the failure to export 3-OH-C_12_-HSL, an *Agrobacterium tumefaciens traG-lacZ* biosensor strain was used to assay signal production in the *adeJ* mutant and wild-type M2 (Niu et al., 2008). However, no significant differences in signal production were observed between these strains (Supplementary Figure 1).

### Role of AdeABC and AdeIJK in biofilm formation

**Figure 2:**
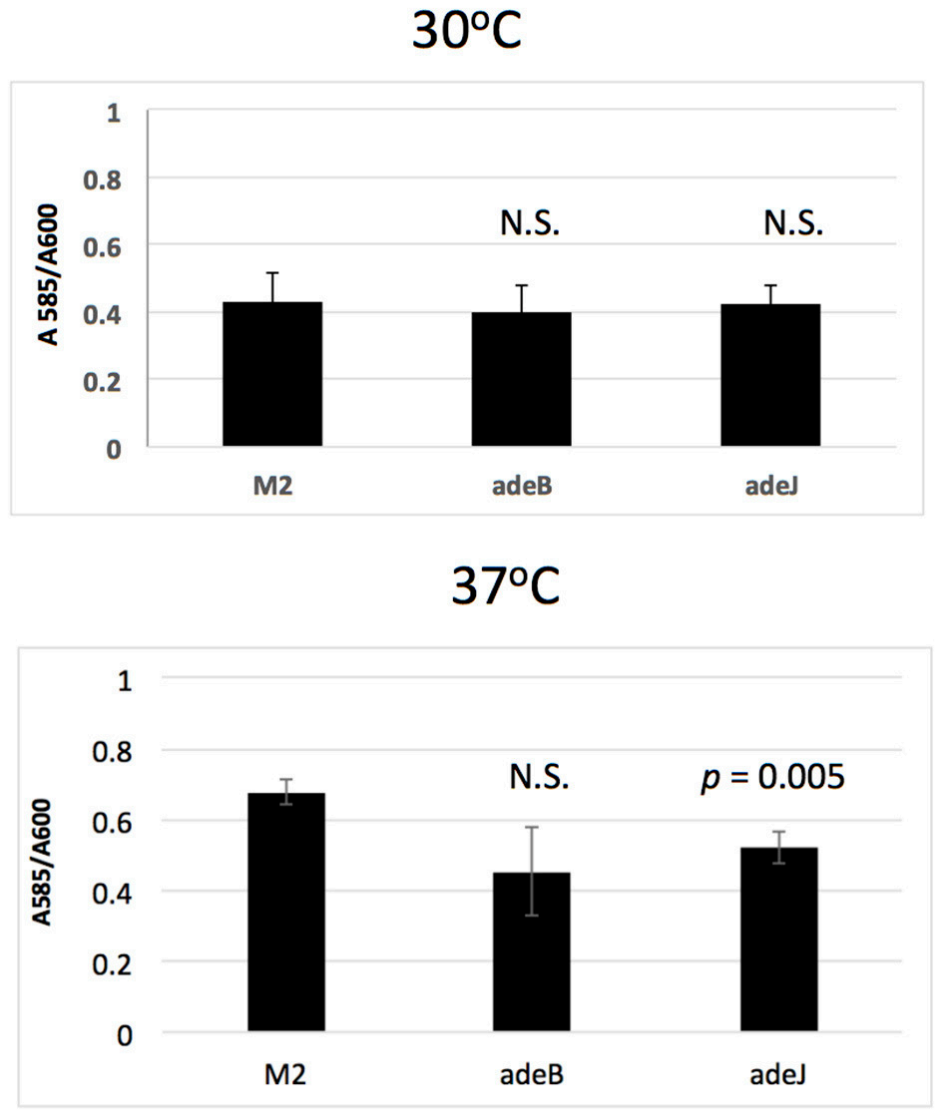
Biofilm formation. Wild-type M2 and the isogenic *adeB* and *adeJ* mutants were assayed for biofilm formation in microtiter wells grown at 30 or 37°C for 24 h. Values represent crystal violet staining/cell density (A_585_ / A_600_) ratio and error bars represent standard deviation of the mean. N.S. indicates a *p*-value > 0.05.

The role of AdeABC and AdeIJK in biofilm formation was also examined. When biofilms were formed on the surface of polystyrene microtiter wells, biofilm formation by the *adeB* and *adeJ* mutants were similar to wild-type M2 after 24 h of growth at 30°C (Figure 2). At 37°C, only the *adeJ* mutant showed a statistically significant reduction in biofilm formation, with a 24% decrease relative to wild-type (Figure 2).

## Discussion

In this study, the roles of AdeABC and AdeIJK orthologs in *A. nosocomialis* were addressed. Both a frameshift allele in the *adeB* gene and an *adeB::Km* disruption did not result in a major change in antibiotic resistance profiles, which is in contrast to that observed in *A. baumannii* (Magnet et al., 2001). Several possibilities can account for these differences. First, the *adeABC* genes may be expressed at very low levels in *A. nosocomialis* M2, therefore, the loss of this efflux system would have a minimal impact. In *A. baumanii*, the AdeABC system is typically expressed at low levels and inactivation of these genes in some strains does not produce a phenotype (Yoon et al., 2015; Leus et al., 2018). Increased expression can result from mutations in the AdeRS two-component system. In *A. nosocomialis* M2, the AdeR and AdeS proteins did not contain amino acid substitutions previously associated with increased AdeABC expression (Marchand et al., 2004; Yoon et al., 2013; Gerson et al., 2018). Based on this information, we propose that the AdeABC genes are tightly regulated by AdeRS and the levels of expression in the M2 strain do not contribute to intrinsic resistance. We also tested the role of AdeABC in both surface motility and biofilm formation and no significant changes were observed in the *adeB* mutant relative to wild-type (Figures 1, 2).

In contrast, the AdeIJK efflux system was shown to play a significant role in antibiotic efflux, where a mutation inactivating this system had a pronounced effect on antibiotic susceptibility (Table 1). This observation is consistent with previous studies in *A. baumannii* demonstrating that efflux mediated by AdeIJK contributes substantially to antibiotic resistance. In addition, the loss of AdeIJK strongly reduced surface motility with a greater than 50% reduction compared to wild-type (Figure 1). The loss of AdeIJK resulted in a modest (24%) reduction in biofilm formation, which is also consistent with previous studies in *A. baumannii*, where the loss of AdeIJK resulted in a 20% reduction in biofilm formation (Yoon et al., 2015). The decreased surface motility and biofilm formation in the *adeJ* mutant were not the result of decreased production of the quorum sensing signal 3-OH C_12_-HSL, which has been shown to be important for both surface motility and biofilm formation in *A. nosocomialis* (Niu et al., 2008; Clemmer et al., 2011).

The mechanism that results in loss of motility when the AdeIJK system in inactivated is unknown, but may indicate a role for AdeIJK in secretion of a lipopeptide surfactant that is required for motilty (Clemmer et al., 2011; Rumbo-Feal et al., 2017) or in the secretion of 1,3-diaminopropane, also required for motility (Skiebe et al., 2012). This also indicates that in addition to antibiotic efflux, there are cellular functions mediated by AdeIJK, indicating a general role for this RND-type efflux system in general physiology of *A. nosocomialis*.

## Author contributions

DK, SR, and PR conducted experiments. PR and RB wrote the manuscript. SR, RB, and PR edited the manuscript.

### Conflict of interest statement

The authors declare that the research was conducted in the absence of any commercial or financial relationships that could be construed as a potential conflict of interest.
