# Supplementary figures and images for: Acinetobacter nosocomialis: Defining the Role of Efflux Pumps in Resistance to Antimicrobial Therapy, Surface Motility, and Biofilm Formation
Source: Front Microbiol. 2018 Aug 21;9:1902. doi: 10.3389/fmicb.2018.01902 (PMC6111201; doi:10.3389/fmicb.2018.01902)

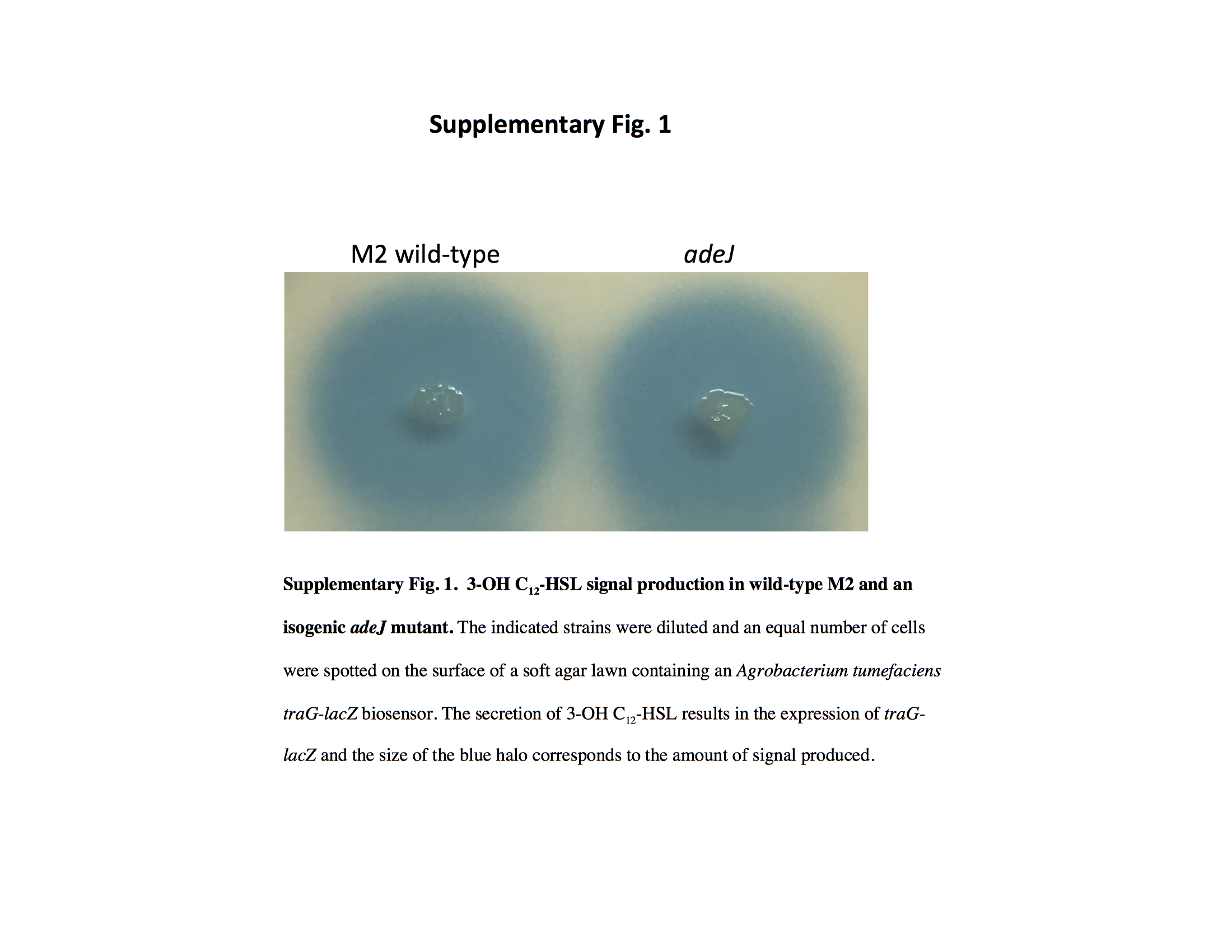

Supplement: Supplementary file 1 [file Image_1.TIFF]
